# Predictors of Negotiated NIH Indirect Rates at US Institutions

**DOI:** 10.1371/journal.pone.0121273

**Published:** 2015-03-20

**Authors:** S. Claiborne Johnston, Susan Desmond-Hellmann, Stewart Hauser, Eric Vermillion, Nilo Mia

**Affiliations:** 1 University of California San Francisco, San Francisco, California, United States of America; 2 University of California Los Angeles, Los Angeles, California, United States of America; Old Dominion University, UNITED STATES

## Abstract

**Background:**

The United States (US) Department of Health and Human Services and the Office of Naval Research negotiate institutional rates for payments of overhead costs associated with administration and space usage, commonly known as indirect rates. Such payments account for a large proportion of spending by the National Institutes of Health (NIH). Little has been published about differences in rates and their predictors.

**Methods:**

Negotiated indirect rates for on-campus research grants were requested from the Council on Governmental Relations for the 100 institutions with greatest NIH funding in 2010. NIH funding, cost of living (ACCRA Index for 2008), public vs. private status, negotiating governmental organization (Department of Health and Human Services or Office of Naval Research), US Census Region, and year were assessed as predictors of institutional indirect rates using generalized estimating equations with all variables included in the model.

**Results:**

Overall, 72 institutions participated, with 207 reported indirect rates for the years 2006, 2008, and 2010. Indirect rates ranged from 36.3% to 78%, with an average of 54.5%. Mean rates increased from 53.6% in 2006 to 55.4% in 2010 (p<0.001). In multivariable models, private institutions had 6.2% (95% CI 3.7%-8.7%; p<0.001) higher indirect rates than public institutions. Rates in the Northeast were highest (Midwest 4.0% lower; West 4.9% lower; South 5.2% lower). Greater NIH funding (p = 0.025) and cost of living (p = 0.034) also predicted indirect rates while negotiating governmental organization did not (p = 0.414).

**Conclusions:**

Negotiated indirect rates for governmental research grants to academic centers vary widely. Although the association between indirect rates and cost of living may be justified, the cause of variation in rates by region, public-private status, and NIH funding levels is unclear.

## Introduction

The US Department of Health and Human Services negotiates payment rates with institutions to reimburse them for facilities and administrative costs related to its contracts and grants; these are commonly called indirect rates. In a US Government Accountability Office analysis of data from the National Institutes of Health (NIH), payments for indirect costs accounted for 28.5% of the extramural budget in 2005, or $4.3 billion [1]. Negotiated indirect rates account for a substantial fraction of institutional support received from NIH. The system for negotiating these rates is complex and expensive, with many institutions paying $1 million or more for consultants in an attempt to increase, or even just maintain, their rates. The Office of Management and Budget (OMB) also consumes substantial resources in negotiating rates, and has recently revised its policies to simplify the process and improve fairness, a common complaint from institutions. The last major attention given to indirect rates occurred in the early 1990’s when questionable expenditures at Stanford were brought to public and congressional attention [2].

In order to better understand the factors that determine institutional reimbursements from NIH, we sought to evaluate predictors of negotiated institutional indirect rates. We obtained indirect rates from institutional surveys and evaluated potential predictors from a number of public sources.

## Methods

Negotiated indirect rates for on-campus research grants were obtained from the Council on Governmental Relations (COGR). COGR members include 150 US research universities, academic hospitals, and foundations with annual federal research grants totaling at least $15 million [3]. COGR surveyed its members in February 2011 requesting the rates for facilities and administration (indirect rates) for federal research from 2006 onward. Given the timing of the survey, rates were only available in 2006, 2008, and 2010, and no survey has been completed since this time.

For this analysis, we included the 100 institutions with greatest NIH funding in 2010. Information on total institutional NIH funding was obtained from the Blue Ridge Institute for Medical Research [4], which analyzes data from the NIH Research Portfolio Online Reporting Tool [5]. Data for total institutional dollars, including all schools, departments, and research programs, were obtained for 2006, 2008, and 2010.

Cost of living for each institution’s local city was obtained from the Council for Community and Economic Research ACCRA Cost of Living Index for 2008, in which 100 denotes average cost among the broadly representative US areas participating and 110 a 10% greater cost than average [6]. Region was classified according to the US Census standards [7]. Public vs. private status was obtained from institutional websites. Negotiating governmental organization was obtained from COGR based on institutional survey responses.

We assessed all institutional variables and year as predictors of institutional indirect rates in univariate and multivariable models. Generalized estimating equations were used to account for clustering by institution over multiple years, with each variable included separately for univariate analyses and with all variables included in the multivariable model. Models were based on a normal function, mimicking linear regression, with an exchangeable correlation structure used to seed the model. Variance in indirect rates were compared by year using Levene’s test, an inferential statistic that tests the hypothesis that variances are equal over multiple groups [8]. Analyses were performed with the Stata software package (Version 13, Statacorp, College Station, TX).

Ethics statement: IRB approval was not required because this study did not involve humans, animals, cell lines, field sampling, or potential biosafety implications.

Competing interest statement: The authors currently or have been employees of institutions reflected in the data.

## Results

Of the 100 research institutions with greatest NIH funding, 72 were COGR members and contributed information about indirect rates in at least one of the three years of study (2006, 2008, and 2010) with 64 contributing information in all years, and with 207 indirect rates reported overall. Indirect rates ranged from 36.3% to 78% over the three years of the surveys, with an average of 54.5% (standard deviation 7.1, 95% confidence interval 46–68%). Total NIH funding ranged from $48 million to $611 million with a mean of $184 million (standard deviation $139 million). Cost of living was greatest in New York City, NY (180.9, equivalent to 80.9% greater than the mean of reporting areas), and least in Houston/Galveston, TX (87.9), with an average of 108.4 among included institutions (standard deviation 24.4).

Among included institutions, 22 were in the Northeast Census region, 20 were in the South, 15 were in the Midwest, and 15 were in the West. Twenty-nine institutions were private and 43 were public.

**Fig 1:**
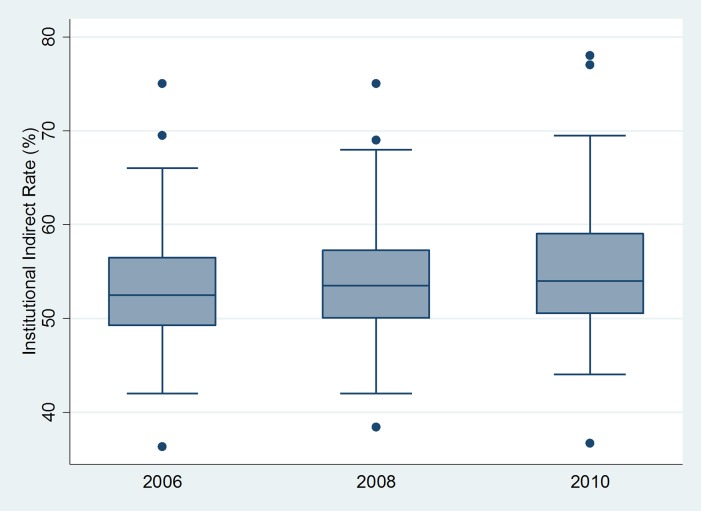
Distribution of institutional indirect rates by year. Boxes encompass 25^th^ to 75^th^ percentiles with whiskers showing nearest adjacent values and outliers displayed as dots.

**Fig 2:**
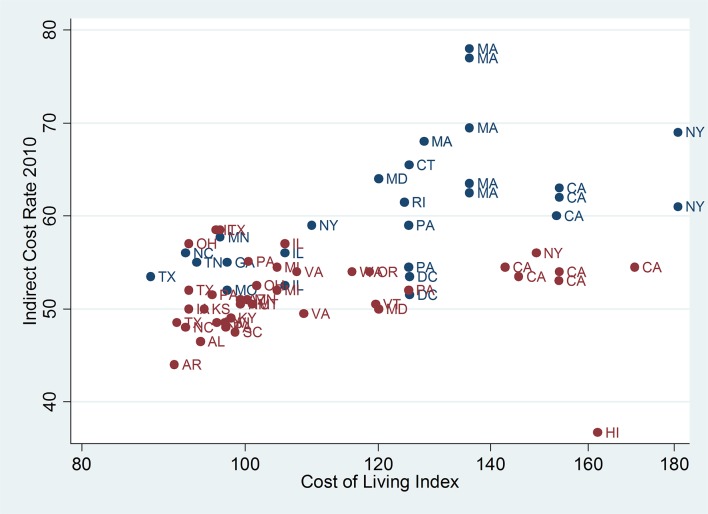
Institutional indirect rates in 2010 by cost of living (ACCRA index). Location of institutions is denoted by standard state abbreviations. Public institutions are shown in red and private institutions are blue.

Several predictors of institutional indirect rates were identified in univariate analyses (Table 1). Indirect rates increased over the study period (from 53.6% in 2006 to 55.4% in 2010, p<0.001) and their distribution remained similarly broad (standard deviation 7.1 in 2006 and 7.3 in 2010, p = 0.978; Fig. 1). Cost of living was a weak predictor in the multivariable model (p = 0.034, Fig. 2, Table 1). The distribution of indirect rates and their relationship to cost of living were similar in all years; thus, only the most recent data from 2010 are shown in Fig. 2. Rates in the Northeast Census region were highest (60.3%) compared to the South (51.0%), Midwest (52.2%) and West (53.3%), and regional differences persisted in the multivariable model (Table 1). Mean rates were 60.0% for private institutions and 50.7% for public institutions (p<0.001), with a persistent difference in the multivariable model (p<0.001; Table 1). Total NIH funding weakly predicted indirect rates in both models (Table 1). Negotiating office within Health and Human Services was not associated with indirect rates (p = 0.414).

**Table 1:** Predictors of institutional indirect rates.

		Unadjusted			Adjusted		
	N*	Mean Rate	(95% CI)	P Value	Rate Diff#	(95% CI)	P Value
Year							
2006	68	53.6%	(51.9%, 55.4%)	Ref	Ref		
2008	72	54.5%	(52.9%, 56.2%)	<0.001	0.9%	(0.6%, 1.2%)	<0.001
2010	67	55.4%	(53.7%, 57.2%)	<0.001	1.8%	(1.5%, 2.1%)	<0.001
Region							
Northeast	63	60.3%	(58.1%, 62.3%)	Ref	Ref		
South	59	51.0%	(49.7%, 52.2%)	<0.001	-5.2%	(-2.0%, -8.5%)	0.002
Midwest	41	52.2%	(51.2%, 53.1%)	<0.001	-4.0%	(-0.5%, -7.5%)	0.024
West	44	53.3%	(51.5%, 55.0%)	<0.001	-4.9%	(-1.7%, -8.1%)	0.003
Institution Type							
Private	85	60.0%	(58.5%, 61.5%)		Ref		
Public	122	50.7%	(50.0%, 51.4%)	<0.001	-6.2%	(-8.7%, -3.7%)	<0.001
Negotiating Entity							
Office of Naval Research	21	56.4%	(53.5%, 59.2%)		Ref		
Health and Human Services	186	54.3%	(53.3%, 55.4%)	0.465	-1.5%	(-5.1%. 2.0%)	0.414
Cost of Living (per 10% increase)	207	1.4%	(0.1%, 0.2%)	<0.001	0.6%	(0%, 1.2%)	0.034
Total NIH Funding (per $100 million)	207	1.3%	(0.2%, 5.8%)	0.024	0.9%	(0.1%, 1.6%)	0.025

*Observations over the three survey periods.

#Rate differences and their 95% CI are shown in absolute terms.

## Discussion

In this analysis of negotiated institutional indirect rates, we found a broad range of rates among institutions, from 36.3% at a public university in Hawaii to 78% at a private hospital in Boston. Rates increased slightly over the 4 years of study with distributions similarly broad. Cost of living accounted for only a small proportion of variability between institutions while other predictors (being a private institution, being in the Northeast US, and having greater NIH funding) were also predictive without a clear explanation.

The association between cost of living and institutional indirect rates may be justified. Cost of living likely impacts direct costs and thus the amount of indirects collected on grants. However, real estate accounts for a large proportion of the variability in cost of living [6] and may be reflected disproportionately in overhead costs encompassed in the indirect negotiations. Nonetheless, it is interesting that the institution with the lowest indirect rate was in an area with one of the highest costs of living (Fig. 2). Furthermore, there were large differences between institutions with similar or even identical costs of living.

It is unclear why US region is independently associated with higher indirect rates. The regional variation alone could result in differences in indirect payments of several million dollars a year between typical institutions in the Northeast and comparable ones in the South, Midwest, or West. Regional differences in building or energy costs seem an unlikely explanation since these are largely reflected in the cost of living index [6].

Institutions with greater total NIH funding negotiated higher indirect rates, with every additional $100 million in total funding associated with a 0.6% absolute increase in institutional indirect rates after adjustment. These differences are not large and may be related to larger institutional investments in consulting, analysis, and negotiation to optimize indirect rates. Alternatively, the higher indirect rates may have led to greater total NIH funding and this alone could have accounted for the association. Regardless, they do not provide evidence of economies of scale.

Private institutions had indirect rates 6.2% higher than those of public institutions after adjustment for other predictors. The cause of this difference is uncertain. Public institutions may receive more governmental support for construction and may leverage state guarantees to negotiate lower interest rates thereby reducing their debt burden, an important component of costs used in negotiating overhead rates [9]. Newer construction and larger investments in equipment may also be more common at some private institutions and these costs may be reflected in the indirect rates. Furthermore, private institutions often invest additional staff time to gather and develop institutional data related to research spending, which can be used to support maximal indirect cost rates.

Other factors not captured in this study could explain some of the differences in indirect rates. For example, space and infrastructure to support laboratory research is more expensive than more traditional office space necessary for many types of population and clinical research, and the relative institutional activity in these types of research was not studied. However, it is unclear how this would explain differences by region or public-private status. In addition, we used a consumer-focused cost of living index [6]. An index that more directly captured costs relevant to research, which was not available, may have explained more variability in indirect rates. Finally, we evaluated only the federal research indirect rate and not the overall rate collected by institutions for the spectrum of their governmental research activities. A prior analysis of reported data from 130 institutions surveyed 1994–1997 suggests that overall rates of collection of indirects are more similar between institutions regardless of their negotiated research indirect rate [9].

A prior analysis of true institutional costs for supporting research suggests that collected indirects generally do not cover all the expenses borne by institutions [9]. Thus, efforts to reduce global indirect rates are likely to have substantial negative consequences in research institutions, which may reduce commitments to research or pass along costs to students [10].

Substantial effort is consumed by academic institutions and also by the government’s Department of Health and Human Services in negotiating indirect rates. Whether this effort produces fairer reimbursement to institutions is uncertain. It may also encourage decisions that reduce the efficiency of the US research enterprise by distracting institutions from gaining additional efficiencies [11,12]. A more extensive study of the intended and unintended consequences of the current policies may be justified, comparing it to a policy that restricts such broad variation in rates. Since the public pays the full cost of research, policies that encourage more efficient distribution of resources could be attractive. Any change from current practices should be staged since it could have substantial financial impact on some institutions.
